# Soil Biodiversity: State‐of‐the‐Art and Possible Implementation in Chemical Risk Assessment

**DOI:** 10.1002/ieam.4371

**Published:** 2020-12-28

**Authors:** Cornelis AM van Gestel, Liesje Mommer, Luca Montanarella, Silvia Pieper, Mike Coulson, Andreas Toschki, Michiel Rutgers, Andreas Focks, Jörg Römbke

**Affiliations:** ^1^ Vrije Universiteit Amsterdam the Netherlands; ^2^ Wageningen University & Research Wageningen the Netherlands; ^3^ European Commission, Joint Research Centre, Ispra Italy; ^4^ German Environment Agency (UBA), Dessau‐Roßlau Germany; ^5^ ERM, Oxford United Kingdom; ^6^ gaiac, Research Institute for Ecosystem Analysis and Assessment Aachen Germany; ^7^ National Institute for Public Health and the Environment Bilthoven the Netherlands; ^8^ Wageningen Environmental Research Wageningen the Netherlands; ^9^ ECT Oekotoxikologie GmbH Flörsheim Germany

**Keywords:** Structural biodiversity, Functional biodiversity, Ecosystem services, Chemical regulations, Protection goals

## Abstract

Protecting the structure and functioning of soil ecosystems is one of the central aims of current regulations of chemicals. This is, for instance, shown by the emphasis on the protection of key drivers and ecosystem services as proposed in the protection goal options for soil organisms by the European Food Safety Authority (EFSA). Such targets require insight into soil biodiversity, its role in the functioning of ecosystems, and the way it responds to stress. Also required are tools and methodologies for properly assessing biodiversity. To address these issues, the Society of Environmental Toxicology and Chemistry (SETAC) Europe 14^th^ Special Science Symposium (SESSS14) was held 19 to 20 November 2019 in Brussels, Belgium. The central aim of the SESSS14 was to provide information on how to include soil biodiversity and soil functions as protection goal options in the risk assessment and quantification of the effects of chemicals and other stressors (including their respective regulations). This paper is based on the presentations and discussions at the SESSS14 and will give a brief update on the scientific state‐of‐the art on soil biodiversity, novel scientific developments, experimental and modeling approaches, as well as case studies. It will also discuss how these approaches could inform future risk assessment of chemicals and other stressors in the regulatory context of protecting soil ecosystems. *Integr Environ Assess Manag* 2021;17:541–551. © 2020 The Authors. *Integrated Environmental Assessment and Management* published by Wiley Periodicals LLC on behalf of Society of Environmental Toxicology & Chemistry (SETAC)

## KEY POINTS

- The SETAC Europe 14th Special Science Symposium (SESSS14) aimed at providing information on how to include soil biodiversity and soil functions (ecosystem services) as protection goal options in the risk assessment and quantification of the effects of chemicals and other stressors.
- Definitions and associated terminology currently used to describe soil biodiversity are given, together with a brief but comprehensive state‐of‐the‐art overview of the current scientific knowledge on soil biodiversity and its relation to the functioning of soils.
- Backgrounds and current practices regarding the way protection of soil biodiversity is addressed in chemical regulations are described, including drawbacks and the possible ways forward.
- Novel methods for assessing biodiversity in soils are presented, as well as approaches, both experimental and modeling, for assessing effects of (chemical) stressors on soil biodiversity and the potential consequences for ecosystem services.

## INTRODUCTION

In its 2030 Agenda for Sustainable Development, the United Nations identified 17 main sustainable development goals (SDGs); in five of these SDGs, protecting the soil is a key element (UN 2015). These subgoals, among others, aim at promoting sustainable agriculture; protecting, restoring, and promoting sustainable use of terrestrial ecosystems; and stopping soil degradation and loss of biodiversity (UN 2015; Keesstra et al. 2016). Soils harbor an enormous diversity, with the highest share in terrestrial ecosystems (Orgiazzi, Bardgett et al. 2016). Soil biodiversity has been shown to be highly important for proper functioning of the soil, for example, to safeguard nutrition and food production (El Mujtar et al. 2019). Food production is one of the ecosystem services provided by soils. Maintaining soil biodiversity may also help prevent outbreaks of pests and diseases and thereby help safeguard human health (Wall et al. 2015). In spite of these factors, which are at the basis of the United Nations SDGs, soil biodiversity is under threat due to intensified land use and associated stressors (Tsiafouli et al. 2015). One of these stressors is chemical pollution, intentionally applied to soil to support crop production (such as plant protection products [PPPs]) or unintentionally ending up in soil by the application of sewage sludge, compost, or manure.

Protecting the biological and physicochemical structure and the functioning of soil ecosystems is one of the central aims of current regulations on chemicals in soil. This is, for instance, shown by the emphasis on the protection of key drivers of ecosystem services as proposed in the protection goal options for soil organisms by the European Food Safety Authority (EFSA 2017). Protecting soil key drivers and the ecosystem services they support requires insight into soil biodiversity, its role in the functioning of ecosystems, and the way it responds to stress. Also required are tools and methodologies for properly assessing biodiversity. To address these issues, the Society of Environmental Toxicology and Chemistry (SETAC) Europe 14^th^ Special Science Symposium (SESSS14) was held in Brussels, Belgium, 19 to 20 November 2019. The central aim of the SESSS14 was to provide information on soil biodiversity and soil functions (both targets of protection goal options) as well as on the effects of chemicals and other stressors (including their respective regulations) on soil biodiversity.

The present paper summarizes the main findings and conclusions of this symposium; in doing so it extends some of the topics presented at the symposium. It will first address the definitions and associated terminology currently used to describe soil biodiversity, followed by a brief but comprehensive state‐of‐the‐art overview of the current scientific knowledge on soil biodiversity and its relation to the functioning of soils. Next, backgrounds and current practices regarding the way protection of soil biodiversity is addressed in chemical regulations will be described, including drawbacks and the possible ways forward. Novel methods for assessing biodiversity in soils will be presented, as well as approaches, both experimental and modeling, for assessing effects of (chemical) stressors on soil biodiversity and their potential consequences for ecosystem services. Finally, the conclusions and recommendations from the presentations and discussions at the SESSS14 will be summarized.

### Soil biodiversity and assessment of anthropogenic stressors

The 3 most important terms of the SESSS14 on soil biodiversity are herein defined as follows:

- Soil biodiversity: The diversity among species of organisms living in the soil and the soil ecosystems of which they are part.
- Structure and function of soil ecosystems: Species belonging to the microbial, micro‐, meso‐, and macrofauna communities, and the ecological functions linked to their activities and interactions.
- Stressors: Anthropogenically induced chemical, mechanical, or biological factors (plus their interactions) that influence soil biodiversity.

Soil ecotoxicology aims to detect, describe, and assess the influence of stressors on the soil organism communities. Historically, this was interpreted as measuring the acute or chronic effects of specific stressors, mainly pesticides, on a limited number of single species, mainly earthworms. Today, the aim is to protect the structure and functions of the (whole) community of organisms, that is, the soil biodiversity. From a regulatory point of view, the new approach has been described in the recent EFSA opinion on soil organisms (EFSA 2017). Another aspect of this shift is the increasing focus on more complex, so‐called “higher tier” (i.e., semifield and field) tests, or the development of new methods such as the DNA‐based identification of individual species in communities. In parallel, modeling of highly complex soil organism communities, both with and without anthropogenic stressors, facilitated their use in regulatory assessment schemes. One recent example is the biodiversity evaluation in retrospective site‐specific assessments (e.g., the Soil Quality TRIAD [SQT] approach) (ISO 2017). So far, these developments strongly focus on temperate regions, but reports from other regions such as the tropics have already been published (e.g., Niemeyer et al. 2015).

Various anthropogenic (e.g., chemical or mechanical) stressors influence soil biodiversity, alone or in combination with “natural” factors such as climate change. Worldwide soil biodiversity is under pressure (Orgiazzi, Panagos et al. 2016), but there is no common approach to addressing stress on soil biodiversity, not in the European Union (EU) or in other parts of the world. Three approaches can be distinguished:

- testing of individual chemicals in a tiered approach, starting with standardized laboratory tests and, potentially, ending up with more complex field studies;
- testing of soil samples collected at more or less contaminated land; and
- site‐specific approaches (e.g., TRIAD).

Despite various regulations that address soil protection in individual Member States of the European Union, soil biodiversity assessment is rarely covered in practice (Ronchi et al. 2019). In contrast, both at the national and the EU level, “classic” soil monitoring is quite common, but it focuses on pedology and chemistry.

## SOIL BIODIVERSITY: SCIENTIFIC STATE OF THE ART

### What is soil biodiversity and why is it important?

Ask people about their associations with the word “biodiversity” and they will probably refer to tropical rainforests, thinking mainly of the aboveground parts of these biomes. What they will not mention is the soil. Yet, a single gram of soil contains more than 1 million taxa, the majority being microbes (Orgiazzi, Bardgett et al. 2016). Soil is thus full of life, with an amazing biodiversity. Soil is the habitat of microbes (bacteria and fungi), microfauna (nematodes and protozoa), mesofauna (microarthropods and enchytraeids), and macrofauna (including earthworms, woodlice, and millipedes). Recently, the global distributions of the abundance and species diversity of several organismal groups were shown in the Global Soil Biodiversity Atlas (Orgiazzi, Bardgett et al. 2016) and in reviews on earthworms (Phillips et al. 2019), nematodes (Van den Hoogen et al. 2019), fungi (Tedersoo et al. 2014), and bacteria (Delgado‐Baquerizo et al. 2018). These publications also illustrate the technical advances in unearthing the diversity of these organisms and their role in the processes for the functioning of our planet (Box 1). Together, soil organisms drive key biogeochemical processes in soil, for instance C and N cycling (e.g., Steidinger et al. 2019), but also determine interactions with the atmosphere (e.g., Wall et al. 2015). Life on earth as we know it is totally dependent on the hidden diversity of organisms living in the soil.

Box 1 **EXPERIMENTAL METHODS FOR QUANTIFYING SOIL BIODIVERSITY PRESENTED AT SESSS14**

Römbke et al. (2018) provide a detailed overview of standardized methods for assessing structural and functional soil biodiversity.

Sampling methods for earthworms combine hand sorting excavated soil cores with extracting animals from deeper soil layers using an irritating agent. For mesofauna, core sampling followed by heat extraction, pitfalls, or suction sampling is used. For microfauna, wet extraction techniques are applied. Animals are identified to different taxonomic levels. Fatty acids (phospholipids [PLFAs] and neutral lipids [NLFAs]) are used as biomarkers of the presence of species, and for assessing microbial species. Recently, DNA (meta‐)barcoding methods are gaining interest for species identification and assessing community composition or the presence of all species in soil samples (soil DNA) (Winding et al. 2019).

Apart from soil biodiversity, the functioning of a soil ecosystem is governed by interactions between species and also with the abiotic environment. Techniques to reveal the trophic structure of food webs include: isotopes ^15^N and ^13^C, PLFA and NLFA biomarkers (PLFA/NLFA), and molecular gut content analysis (DNA) to reveal prey species profiles. These techniques provide novel insights into (effects on) species interactions and the role in processes such as the turnover of nutrients.

Tests on functional endpoints, that is, the processes performed by the soil community, include the litter bag test (decomposition) and the bait lamina assay (soil fauna feeding activity). A range of microbial functional tests are available, including the nitrification test, multienzyme assays, and catabolic profiling bioassays.

Nowadays, there is great concern about the worldwide decline in biodiversity and the consequences for ecosystem services (Cardinale et al. 2012). The insect decline has received a lot of attention recently (Hallmann et al. 2017), but similar concerns also exist for soils: A decline in soil biodiversity leads to decreased soil functioning (e.g., Orgiazzi, Panagos et al. 2016). A comprehensive assessment of land degradation and restoration by the Intergovernmental Platform for Biodiversity and Ecosystem Services (IPBES 2018) has documented that land degradation affecting belowground biodiversity across the globe is a widespread and severe issue and shows no signs of slowing down. This trend must be halted and reversed (e.g., Willemen et al. 2020). The Global Soil Biodiversity Initiative (GSBI) is federating recent efforts to reverse the current trend of soil biodiversity loss and is providing fully updated information on global and regional data and scientific evidence on soil biodiversity (https://www.globalsoilbiodiversity.org/).

In the past, the field of ecotoxicology had not really embraced the myriad of soil life for soil functioning, given that ecotoxicological tests were tailored mainly to single species, such as earthworms, enchytraeids, and springtails. Clearly single‐species tests have limited power when explaining effects on the highly diverse, complex soil communities in the field and their relationships with ecosystem services (Faber et al. 2019).

### Vital soil functions

Soil biodiversity is vital for food production, regulation of clean environments (including water), cycling of nutrients, and climate change mitigation. The importance of soil for sustainable living on the planet is illustrated by the fact that “soil” is mentioned in 5 of the 17 SDGs (UN 2015). Soil biodiversity is underlying these goals but also has an intrinsic value in itself (IPBES 2018).

Habitat fragmentation, climate change, intensive human exploitation, organic matter decline, soil compaction, soil erosion, salinization, and pollution are all recognized as potential threats to soil biodiversity (Orgiazzi, Panagos et al. 2016). Intensive land management, characterized by high N inputs, deep ploughing, and high grazing, appears to be the major threat for biodiversity loss. Soils under pressure were often located outside nature management areas (i.e., in agricultural hot spots).

Collaborative research projects, such as EcoFINDERS (2011–2014) (https://projects.au.dk/ecofinders/), gave insight into soil biodiversity across Europe. Studies on the relationships between agricultural management and soil biodiversity showed that agricultural intensification has resulted in a decline in soil C, biological activity, and soil biodiversity (Tsiafouli et al. 2015). The complexity of the interactions among different functional groups of soil organisms—and the changes therein due to land use—was enormous. Understanding changes at low taxonomic levels (so, at high taxonomic resolution) are demanding and need new techniques such as next‐generation sequencing (Box 1), but progress has been made on the basis on lower scales of resolution. Earthworms and arbuscular mycorrhizal fungi are often well represented in soil food‐web models, but saprophytic fungi, protists, and bacteria are underrepresented and often only their biomass ratio is reported (Grigulis et al. 2013). Soil respiration increased with increasing earthworm biomass and fungal‐to‐bacterial ratios, but N cycling was enhanced if arbuscular mycorrhizal fungi and bacterial biomass were high (De Vries et al. 2013). An even lower scale of resolution would define C content as a key proxy for yield prediction, optimal fertilizer use, and biomass of the soil food web (Birkhofer et al. 2008).

The project LANDMARK (www.Landmark2020.eu; Schulte et al. 2015) developed a semiquantitative assessment system for 5 overarching soil functions (primary production, water quality and quantity, nutrient cycling, climate regulation) and for soil biodiversity. A decision support system, based on quantitative criteria, was built and tested, where integrated information that defines soil biodiversity was scored on a scale from low to medium to high (www.soilnavigator.eu; Debeljak et al. 2019). Soil management and climate were the main predictors for soil biodiversity, which is interesting in the context discussed at the symposium, because it may provide an opening for linking the effects of PPPs and other strategies against pests into a sustainable soil management system. This potential link may allow for assessing the capacity of soils to deal with different stressors, including chemicals as pesticides.

### Interactions with plants

Plants interact with the soil and with other soil organisms via their roots (Bardgett et al. 2014). Specifically, plants can shape the composition of soil communities, with feedback on their growth (Bever et al. 2012) and nutrient cycling (Chen et al. 2017; Jílková et al. 2020). This plant–soil feedback over long time periods is also referred to as “legacy effect” (Kardol et al. 2007), and often increases in strength over time (Eisenhauer et al. 2012). Legacy effects are likely different for organisms of different size and mobility (Scherber et al. 2010). Decaying roots (Mommer et al. 2015; Chen et al. 2017), root respiration rates, C investments to mycorrhizal symbiosis (Bergmann et al. 2020), and root exudates that fuel bacteria biomass provide major sources of C to the soil. Linking these plant traits to the functioning of soil organisms is an important next step to be made to understand soil functioning and soil biodiversity (Bardgett et al. 2014).

Direct effects of (chemical and nonchemical) stressors on soil biota may be fundamentally different from indirect or cascade effects, such as the plant‐induced legacy effects or effects of small changes in the soil food‐web structure over time. In the case of short‐term, high‐level exposure, stressors may provoke an acute effect, killing or affecting part of the soil community. In the case of long‐term chronic exposures to low levels of stressors, the potential resilience of soil communities or their components may be impaired (Brock et al. 2018). In all cases, it is important to note that soils are dynamic and interlinked systems, with strong feedback between the components of soil communities, making it necessary to embrace simplifications and define more easy‐to‐use criteria for soil biodiversity.

## SOIL BIODIVERSITY PROTECTION GOALS IN GLOBAL FRAMEWORKS AND CHEMICAL REGULATIONS

Several legislative frameworks aim to protect ecosystems. It has been broadly recognized that the diversity of life at all levels of biological organization needs to be protected for its intrinsic value and for the services it provides to humanity (e.g., MEA 2005). Accordingly, protection goals for biodiversity at large are set in communications, resolutions, and common strategies in Europe and worldwide, such as the EU Biodiversity Strategy (EU COM 2011) and the United Nations SDGs (UN 2015). Additionally, the vision is that the natural capital in the European Union, and the ecosystem services it provides, are by 2050 “protected, valued and appropriately restored for biodiversity's intrinsic value and for their essential contribution to human wellbeing and economic prosperity.” The role of agricultural systems and their management for the protection of biodiversity is further acknowledged because sustainable agricultural practices that protect biodiversity, improve the status of species in agricultural landscapes, and provide ecosystem services are explicitly promoted. All these elements have been introduced in the recently proposed European Green Deal, especially within the new Biodiversity Strategy for 2030, Farm to Fork, and the so‐called “Zero‐Pollution Action Plan” (Montanarella and Panagos 2021).

At the global level, the UN Convention on Biological Diversity has established targets to reach the protection of biodiversity (Aichi Targets, UN 2015). In addition to the overarching strategic goal to improve the status of biodiversity by safeguarding ecosystems, species, and genetic diversity, a specific focus is on managed systems, in order to address the underlying causes of biodiversity loss and to reduce the direct pressure on biodiversity. By 2020, for instance, “areas under agriculture, aquaculture and forestry are managed sustainably, ensuring conservation of biodiversity” and “pollution has been brought to levels that are not detrimental to ecosystem function and biodiversity.”

With the aim of avoiding unacceptable effects of chemical pollution, protection goals for biodiversity are also included in the legislative framework of regulated products. Regulation 1107/2009 for placing PPPs on the European market (EU COM 2009) and the European Biocidal Products Regulation (EU COM 2012) define conditions of approval for products. To be authorized, a product “shall have no unacceptable effects on the environment, having particular regard to…its fate and distribution in the environment…, its impact on non‐target species, including on the ongoing behavior of those species; and its impact on biodiversity and the ecosystem.”

However, even if halting biodiversity loss (also in managed systems) is a key target in the European Union and at the international level, and provisions for chemical authorization exist to exclude “unacceptable impacts on biodiversity,” the successful implementation of biodiversity protection goals in everyday risk assessment procedures is currently not straightforward.

To define more specific parameters of assessment (e.g., magnitude, temporal and spatial scale of tolerable effects), EFSA has identified specific protection goal (SPG) options for soil organisms, tackling soil biodiversity and ecosystem services driven by soil ecosystems (EFSA 2017). Because the ecosystem services concept (MEA 2005; EFSA 2016) considers biodiversity and species abundance as the motor for delivering ecosystem services, it was explored by EFSA how to achieve the goal of protecting biodiversity itself within the ecosystem services concept. The following components of biodiversity “necessary” for in‐soil organisms in agricultural landscapes were identified and should be tackled in assessment frameworks:

- the biodiversity of in‐soil organisms per se, that is, the “intrinsic” value of biodiversity as a regulated good that needs to be provided;
- the performance of ecosystem services driven by in‐soil organisms, with particular reference to changing environment and multiple stressors, that is, the supporting services of diverse soil communities for the functioning of soils; and
- an option value for biodiversity and genetic resources in the long term, that is, the provisioning service to be able to take advantage of ecosystem services driven by the soil organism community now and in the future.

As noted by Cardinale et al. (2012), maintaining multiple ecosystem processes at multiple places and times requires higher levels of biodiversity than a single process at a single place and time. Therefore, care should be taken in assessing single ecosystem services and assuming that by protecting current functional levels biodiversity is also maintained. In such cases, species erosion might take place to the point at which service level changes occur (tipping points of ecosystem functioning), indicating unsustainable soil protection.

## ASSESSING CHEMICAL IMPACTS ON SOIL BIODIVERSITY UNDER GLOBAL FRAMEWORKS

The components of the soil ecosystem to be assessed in relation to the assessment of the impact of PPPs or other chemicals at the global level are determined by the definition of sustainable soil management from the Revised World Soil Charter (FAO 2015): Soil management is sustainable if the provisioning, regulating, and cultural ecosystem services provided by soil are maintained or enhanced without significantly impairing the soil functions that enable those services. Therefore, the application of PPPs or other chemicals conflicts with the aim of sustainable soil management if their use significantly impairs either 1) soil functions or the ecosystem services provided by those functions or 2) biodiversity.

An extensive assessment, at the global level of the impact of PPPs on soil functions and soil ecosystems, has been completed by the Intergovernmental Technical Panel on Soils (ITPS) (FAO and ITPS 2017). This assessment builds upon previous initiatives of the ITPS and the Global Soil Partnership (GSP). The Revised World Soil Charter (FAO 2015) establishes a definition for sustainable soil management that can be applied to the assessment of PPPs at global levels. The definition of PPPs used in the FAO and ITPS (2017) assessment is as follows: “Plant protection product means a pesticide product intended for preventing, destroying or controlling any pest causing harm during or otherwise interfering with the production, processing, storage, transport or marketing of food, agricultural commodities, wood and wood products.” Specific legislative frameworks are in place for the risk regulation (assessment and management) of PPPs in, for example, the European Union, the United States, Canada, and Brazil, which need to be followed for the approval of active substances or the authorization of PPPs (see section on *Current practices regarding soil biodiversity protection in chemical regulations*). The Status of the World's Soil Resources Report (FAO and ITPS 2015) synthesized current knowledge about a key component of the assessment, soil biodiversity, and soil contamination. Finally, the Voluntary Guidelines for Sustainable Soil Management (FAO 2017) provide guidance on sustainable soil management practices.

## CURRENT PRACTICES REGARDING SOIL BIODIVERSITY PROTECTION IN CHEMICAL REGULATIONS

Although there are regulatory requirements for assessing the safety of chemicals for soil ecosystems in various countries and with respect to various chemical types, the most advanced regulatory assessment scheme is in the European Union for PPPs. The current scheme, with reference to European Commission regulation 1107/2009 (EU COM 2009), is predominantly aimed at the preservation of communities of soil organisms in the in‐field area. Within the current European data requirements for PPP evaluation (EU 283/2013 [EU COM 2013a] and 284/2013 [EU COM 2013b]), the majority of requirements for soil organisms are for single‐species toxicity tests, although higher tier (field) studies can investigate effects on populations and communities (see also EFSA 2017). Another nuance with the current regulatory scheme is that it conducts risk assessments within compartments (e.g., soil or water) or species groupings (e.g., nontarget arthropods and soil fauna). Some species however, are found within more than 1 compartment due to changes during their life cycle: For example, a nontarget carabid beetle may be predominantly a soil dweller as a larva and pupa but then mostly a surface dweller as an adult. The compartmentalization of organisms within the risk assessment scheme currently, while a pragmatic approach, does not lead easily to assessing the effects on overall biodiversity (even when this term is defined for EU regulatory purposes). Nevertheless, it appears intuitive that if acceptable acute and chronic risks are concluded for each set of organisms, then this approach also has to be directionally correct in terms of protecting soil biodiversity.

Studies currently needing to be performed within the first step (or tier) of the risk assessment for soil organisms in the framework of the European regulation on PPPs (EU COM 2009) include earthworms, collembolans, soil mites, and soil microflora (functional test only). Several nontarget arthropods can be seen as soil organisms (at least in parts of their life histories) and are also addressed, but within the nontarget arthropod assessment. Earthworms, collembolans, and soil mites are considered together because their testing and risk assessment approaches are virtually identical. Initial toxicological laboratory studies are conducted to derive measurement endpoints as effect concentrations (ECs) at which effect levels are detected (e.g., EC10, EC20, EC50) or as no observed effect concentration (NOEC), based mostly on reproduction outputs but also including bodyweight for earthworms. These endpoints are then compared to the maximum predicted environmental concentration for soil (PEC_SOIL_) to derive a toxicity exposure ratio (TER). The TER is compared to a pass/fail criterion of 5 and then either an acceptable risk is concluded or higher tier studies, risk assessments, or modeling can be conducted to indicate acceptable risks.

Soil microflora is currently addressed only with a functional test investigating N transformation. The risk is not addressed by a TER approach but needs to pass the following criterion: less than or equal to a 25% effect after 100 d of exposure (when compared to the control).

Currently there are no well accepted “interim tier” or semifield studies; therefore testing strategies go from standard tests with single species to the field‐testing level. For each group of organisms, changes are measured and compared to an untreated control and a toxic standard or reference substance. Individual species, species groupings, and ecological niches (e.g., epigeic and endogeic species in the case of earthworms) as well as total numbers can all be investigated in the field. The regulatory criterion is the time until recovery to control levels at the same time point, given that population numbers can go up and down throughout the year.

To cope with the lack of an interim tier and to meet the requirement of the European regulatory framework for PPPs (EU COM 2009) to explicitly consider impacts on nontarget species, their ongoing behavior, biodiversity, and the ecosystem, including potential indirect effects via alteration of the food web, there is a need for semifield studies, potentially considering communities of species. Several mesocosm tests have been developed, using assemblages of selected species. Such mesocosm tests may allow for assessing effects on the interaction between the chosen species; however, they are not sufficiently capable of assessing effects on soil biodiversity. A more complex but also more effective method, which has been proven to be feasible to address these issues and has already been proposed as a potential higher tier test system for a refined risk assessment, is a terrestrial model ecosystem (TME) (Schäffer et al. 2010; Box 2). In addition, ecological models develop toward a serious instrument for the prospective risk assessment. Ecological models can be fed ecotoxicological results from first tier experiments and validated with higher tier, for example, TME experiments. They can be used to extrapolate laboratory‐based findings across temporal and spatial scales, and to screen exposure situations in different soils and under different conditions (e.g., pH, temperature) than used in laboratory tests (see section about *Soil biodiversity and modeling of chemical effects*).

Box 2 **TERRESTRIAL MODEL ECOSYSTEMS**

Terrestrial model ecosystems (TMEs) are available in 2 versions. Field TMEs are situated outdoors, exposed to natural weather conditions, and contain intact soil cores from untreated grasslands with a natural soil community of species. Laboratory TMEs typically are smaller and allow control of test conditions, but often cannot be maintained for more than 4 to 5 mo. A dose–response design is chosen for TME studies with at least 5 replicates per treatment.

Fate and effect of chemicals are usually monitored in outdoor TME systems over a period of 1 y. The recommended taxonomic groups studied in TMEs are the most abundant representatives of the soil mesofauna. Except for earthworms, the sampling is performed by taking subsamples from each TME at each sampling date according to specific density and variability of each taxon. For earthworms, the whole TME has to be sampled destructively at each sampling date. After extraction, the organisms are counted and determined to the species or family level. Univariate and multivariate statistical analyses are applied to assess effect concentrations or thresholds on population and community levels (Toschki et al. 2020).

In TMEs, the fate of pesticides and their effects on soil organisms and soil communities can be investigated in space and time. Impacts on natural communities, including changes in behavior, species interactions, and diversity, can be evaluated under realistic conditions over 1 y. Using outdoor TMEs allows the testing of different environmental scenarios (drought, multiple application, mixture toxicity) on the community level.

## ADVANCEMENTS IN EFFECT ASSESSMENT AND RISK CHARACTERIZATION FOR SOIL BIODIVERSITY

The EFSA opinion on in‐soil organisms (EFSA 2017) identifies several gaps and developments needed to advance the effect assessment and risk characterization for soil organisms and soil biodiversity when exposed to chemicals, here, PPPs. Besides possible gaps in the coverage of species or organism groups by the standard tests and some methodological problems, more general developments also were called for. The test battery according to data requirements of the European regulations 283/2013 (EU COM 2013a) and 284/2013 (EU COM 2013b) was assessed to be sufficient to cover the effects of PPPs on soil organisms, with the exception of organisms exposed mainly via food (e.g., predators), litter‐dwelling species (e.g., isopods), and microorganisms (i.e., the lack of effect testing on soil fungi). Uncertainties exist regarding the correct assessment of chemical effects on soil biodiversity in the field, on the one hand because of methodological and statistical problems, and on the other hand because of missing guidance to evaluate impacts on soil biodiversity apart from effects on earthworm communities. When assessing chemical effects on soil biodiversity in the field, it was deemed central to assess whole communities and not to separate the evaluation for single groups as is currently done (e.g., earthworms, microarthropods), thereby missing indirect effects and the impact on species interactions.

In order to fully address chemical effects on soil biodiversity, reference systems should be chosen ideally to present an identical system but without the stressor. Two issues are eminent. First, each system including the reference is shaped by a unique set of environmental conditions; only one of them is related to chemical stressors. Consequently, the difference between impacted and reference systems is the result of the chemical impacts plus that of confounding factors. Second, each system exhibits variations in space and in time, which cannot be attributed to environmental factors or chemical effects. It has been argued that so‐called “normal operating ranges” (NORs) for natural and managed systems should be explored. In this context, NOR should be used to define the normal variation of soil biodiversity in a certain area which cannot be attributed to stressors or other environmental factors. Deviations from NOR show adverse effects that might be due to, for instance, chemical stressors. Soil biodiversity protection and sustainable, multiple service provision should be assessed not only under more realistic conditions of use, for PPPs namely not as single product, single use, but also under year‐on‐year, long‐term spray series of a suite of (different) products, possibly under the impact of additional stressors in the agricultural system at stake. Improving the reliable detection of chemical effects on (the different components) of soil biodiversity would reduce existing uncertainties regarding the assessment of chemical impacts on soil organisms and on the correct implementation of the general protection goals for regulated products, which should have no unacceptable effects on biodiversity and the ecosystem.

## OUTLOOK: THE FUTURE OF DESCRIBING SOIL BIODIVERSITY

In addition to the highly standardized laboratory tests, higher tier studies and tests with functional endpoints have been developed, but with the exception of earthworm field tests (ISO 1999) they are rarely used. However, current developments indicate a growing interest in soil biology, for example, the monitoring of soil microbial communities or even the distribution of soil functions in Europe (e.g., Thomson et al. 2015; Van Leeuwen et al. 2017). The European guidance on PPPs does trigger higher tier earthworm, microarthropod, and litterbag tests, and also includes pesticide fate to address exposure. Still the best‐known approach to include soil biology in site‐specific soil assessments is the TRIAD methodology (ISO 2017) (Box 3).

Box 3 **SOIL QUALITY TRIAD: ECOLOGICAL RISK ASSESSMENT TOOL SUPPORTING SOIL MANAGEMENT AT CONTAMINATED SITES**

**Figure 1:**
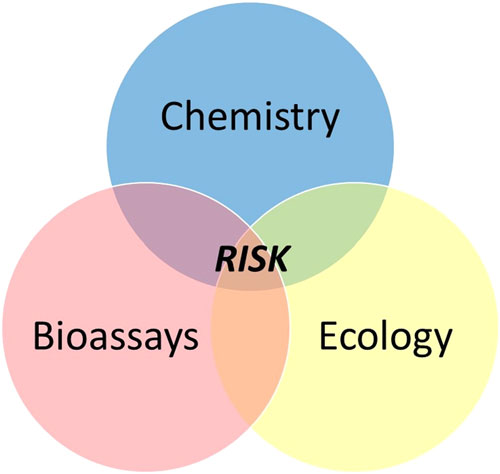
Three lines of evidence of the Soil Quality TRIAD approach.

The Soil Quality TRIAD (SQT) is a tool to assess site‐specific ecological risks at contaminated sites (Swartjes et al. 2012), simultaneously deploying 3 lines of evidence (LoE) (Figure 1): Chemistry = chemical characterization, Bioassays = toxicity characterization, and Ecology = ecological surveys.

In the standardized SQT (ISO 2017), chemical characterization is accomplished through calculation of the toxic pressure (TP), using concentration and response addition models for mixture toxicity. To synchronize with the other LoEs (toxicity, ecology), the TP is calculated from species sensitivity distributions with EC50 data. The toxicity characterization uses simple and standardized bioassays. The ecological field may include a vegetation analysis, but also soil‐dwelling organisms such as the nematode community.

By adding the 3 LoEs to a weight of evidence (WoE), uncertainty reduction is accomplished and decisions for the management of the contaminated site can be based on this information. A trigger for delineating the information is the “deviation factor,” quantifying the level of disagreement between the LoEs (Rutgers and Jensen 2011).

To extrapolate results from the SQT, a standard curve linking the TP in the chemistry LoE to the integrated risk value of all LoEs is applied, allowing the assessor to accept a lower precautionary safety factor. This approach may reduce the required management for the contaminated site. For easy interpretation of SQT results, presentation schemes have been developed (Rutgers and Jensen 2011; Niemeyer et al. 2015).

One precondition for performing wide‐range soil biological monitoring programs, especially in the context of assessing and regulating soil quality, is the use of standardized methods. So far, a standard method for determining the microbial diversity in soil is available (ISO 2011). This standard has already been used for the evaluation of soils in a Europe‐wide project on the assessment of biological soil quality (Plassart et al. 2012). A general overview on existing standards (and the respective gaps) is given by Römbke et al. (2018) and, with a special focus on microbial functional methods, by Thiele‐Bruhn et al. (2020). Novel methods such as environmental DNA (eDNA) for assessing biodiversity and tools for assessing interactions between species in the terrestrial food web (Box 1) also need to find their way into the assessment of stressor effects on soil biodiversity.

Another concept is that effects of chemical stressors heavily interact with the effect of other factors that influence structural and functional soil biodiversity. Geisen et al. (2019), for instance, indicated that human impact, including pollution and its side effects, does lead to reduced soil biodiversity, food‐web stability, resilience, and sustainability of the soil, whereas the risk of pathogens, dependency on management, and costs of land use increase.

Finally, a proper assessment of chemical (and nonchemical) stressor effects on complex systems like soil communities can benefit greatly from the use of modeling approaches (see section *Soil Biodiversity and Modeling of Chemical Effects*).

## SOIL BIODIVERSITY AND MODELING OF CHEMICAL EFFECTS

As mentioned in the section *Soil biodiversity: Scientific state of the art*, soil biodiversity is high, and the description of soil communities is rather challenging, not least because soil communities are extremely complex and diverse, with huge numbers of interacting species and individual organisms. Modeling complete soil communities is therefore challenging, and a clear target for such modeling efforts is needed. One possible viewing angle on modeling soil communities is the theoretical analysis of bioenergetic consumer‐resource models, including species trophic interactions (e.g., Brose et al. 2005). Such techniques enable the formulation of complex community structures in a generic way, and allow for stability analyses and the exploration of ecological theories. Nevertheless, the parameterization of such models is usually not specific for real soil organisms, and the number and complexity of the food webs is still smaller than in real soils. With respect to chemical risk assessment, such modeling approaches are not specifically useful because the “species” in such community models cannot be connected to specific sensitivities to chemicals. Another direction for modeling soil communities is the functional view; functional models are often used to describe the flux of nutrients as C, N, or P in soil (e.g., DNDC, Expert‐N, Roth‐C; an excellent directory of functional and soil nutrient flux models is available on the website of the International Soil Modeling Consortium: https://soil-modeling.org/resources-links/model-portal).

The active part of soil communities in such nutrient turnover models often is restricted to microbial biomass, and trophic interactions with other groups in soil are not included. In some cases, nutrient turnover models have been applied for the evaluation of effects of chemicals such as antibiotics in soil (e.g., Schauss et al. 2009). Still in early stages are attempts to construct soil communities and food webs automatically by artificial intelligence or machine learning techniques (e.g., Bohan et al. 2011). Such approaches could be empowered by recent advances in molecular biology, but they also would have the downside of not being transparent and of not yet being ready to use in regulatory risk assessment.

Besides soil community and food‐web modeling approaches, another and probably currently most developed approach is the mechanistic modeling of soil keystone species. Mechanistic models are available for major keystone species, particularly for earthworms. Individual‐level models for the assessment of chemical effects on survival, growth, and reproduction of earthworms were developed in the early 2000s, based on an explicit description of toxicokinetic processes that lead to internal concentrations (e.g., Jager et al. 2003). Using energy budget modeling, models could be calibrated to account for chemical effects of certain substances. At population levels, Johnston et al. (2015) developed the Energy–Environment–Earthworm (EEEworm) modeling framework, in which individual‐based models are used to model growth, reproduction, and behavior of earthworms based on energy budgets, in connection with spatially explicit movement in interaction with a dynamic environment. The EEEworm model was calibrated to represent *Eisenia fetida*, the standard organism for ecotoxicological experiments, but the model has also been parameterized for the endogeic species *Aporrectodea caliginosa*, and a trade‐off situation between preferred soil moisture conditions and food availability could be explored. The EEEworm model framework was also used to analyze effects of pesticides and other agricultural management practices (Johnston et al. 2015). Currently, further developments of the EEEworm modeling framework into a fully functional spatiotemporally explicit toxicokinetic–toxicodynamic model for earthworm toxicity (Roeben et al. 2020) aim to couple spatially explicit exposure information with individually moving earthworms for the use in future chemical risk assessment. Challenges to be solved are multifold, not only for the modeling but also for questions such as how representative the standard test species *E. fetida* is for other, potentially more sensitive earthworm species.

## CONCLUSIONS AND OUTLOOK

The SESSS14 on soil biodiversity resulted in the following main overall conclusions and recommendations:

Approaches to investigate (effects of anthropogenic stressors on) multispecies interactions should be developed and could involve both testing and modeling. There is value in further single‐species testing, for example, to close gaps in knowledge and to understand mechanisms of intoxication. But single‐species tests have limitations. It might perhaps be considered to include different soil organism species exposed at the same time, using mesocosm designs such as the TMEs (Box 2), because this may help understanding interactions in the soil communities and their response to chemical and other stressors. Additionally, modeling approaches could help to extrapolate single‐species tests beyond the restrictions of the specific laboratory testing conditions.

It is realized that exposure to a (single) chemical stressor only is rare; rather there will be exposure to multiple chemicals and other stressors. As a consequence, there is a widespread interest in going beyond single‐chemical testing to increase realism, including parameters such as chemical persistence, bioaccumulation, and interactions between chemical and other stressors. Such a multiple stressors approach could involve, for example, testing and accounting for dynamic mixture exposures and multiple stressor interactions. Again, modeling approaches provide the possibility to simulate the effects of exposure to multiple chemicals by mixture effect modeling in combination with population models. In addition to mixture toxicity simulations, ecological models can also account for other stressors, such as deviations from preferred temperature ranges or changes in soil structure or water content if the required ecological knowledge is available and implemented in the model. In that way, nonchemical stressor impacts can be coupled with the impact of single or multiple chemicals.

The overview of our current knowledge on soil biodiversity has demonstrated the importance of time, for example, in the legacy effects seen for the interaction between aboveground and belowground components of soil ecosystems or in the toxicokinetics and toxicodynamics of chemicals. It therefore is recommended to acknowledge the effect of time in ecological processes by taking into account long‐term exposures, development of (multiple and dynamic) exposure levels, effects over multiple generations, the occurrence of delayed effects, and the consideration of explicitly dynamic modeling approaches.

Projects at the European level (e.g., EcoFINDERS and LANDMARK) have shown the importance of regional differences in the assessment of soil biodiversity and its response to chemical and other stressors. It therefore is recommended to account for different environmental factors, for example, different climatic zones, soil types, and land‐use practices, when studying the interaction of stressors and soil biodiversity.

It is obvious that the complexity of the soil community and its interactions does not allow for an easy prediction of ecotoxicological effects on soil biodiversity and its functioning in ecosystem services. Apart from investing in novel tools for assessing effects on structural and functional components of soil biodiversity, recent developments in modeling could help with all the aspects of extrapolating from single species effects to impacts on structural and functional components of soil biodiversity and should be included early in the process of risk assessment. Modeling could also help predict effects at the landscape level and predict long‐term implications, or use ecological information to link exposure and effects, and ultimately also to connect service‐providing units to ecosystem services in the ecosystem services concept as proposed by EFSA.

The discussions at the SESSS14 on soil biodiversity, and also the drafting of elements in the present paper, show that building a “watertight” precautionary approach to multiple stressor impacts on soil biodiversity and ecosystem services is challenging. Several examples from the past have shown that regulatory implementation of scientific developments in risk assessment is running behind developments in chemistry. As such, a postregistration monitoring approach may help inform on the impact of chemical and nonchemical stressors on soil structure and functions, in a way that cannot easily be included in an initial registration process but contributes to the identification of upcoming risks.

There also is a need for further development of novel methods for assessing structural biodiversity, such as eDNA methods for identifying species diversity in addition to and/or alternatively to traditional morphological species identification. Similarly, new functional methods, especially addressing soil microbiology, are needed to enable the proper assessment of effects of stressors on biodiversity and its role in ecosystem services. Such improvement could also enhance understanding the link between biodiversity and ecosystem services and in this way support the development of specific protection goals for soil organisms (e.g., as proposed by EFSA) and the United Nations SDGs.

## Disclaimer

The authors declare no conflicts of interest.

## Data Availability

Data are available upon request from corresponding author Cornelis AM van Gestel (kees.van.gestel@vu.nl).
